# Genetic and root phenotype diversity in Sri Lankan rice landraces may be related to drought resistance

**DOI:** 10.1186/s12284-016-0092-7

**Published:** 2016-05-17

**Authors:** Mayuri Munasinghe, Adam H. Price

**Affiliations:** 1 Institute of Biological and Environmental Sciences, University of Aberdeen, Aberdeen, AB24 3UU UK; 2 Current address: University of Sri Jayewardenepura, Nugegoda, Sri Lanka

**Keywords:** *Oryza sativa*, Rhizotron, SNP, Structure, Herbicide, Indica

## Abstract

**Background:**

The development of relatively cheap and high throughput methods of genotyping and phenotyping plants offers the opportunity to explore local germplasm more thoroughly than before and should accelerate the identification of sources of genetic variation suitable for breeding. In this study, 135 Sri Lankan accessions, mostly identified as landraces, for which data was available at the International Rice Research Institute on drought scores were genotyped using a 384 SNP array and assessed for root depth using a newly developed buried herbicide method. Roots of 36 accessions were assessed using hydroponics and 12 using soil-filled rhizotrons to establish if variation in herbicide score could be attributed to root traits.

**Results:**

Population structure based on the SNPs using STRUCTURE revealed six groups, being *tropical japonica*, *aus* and four *indica* subpopulations. Three of these *indica* subpopulations do not seem to be represented in the Rice Diversity Panel I (RDP1) of 372 global rice accessions and appear to represent genetic diversity so far poorly studied by the global scientific community. The herbicide score was highly discriminatory between landraces and correlated very strongly with hydroponic and rhizotron root traits. The mean herbicide score strongly differentiated between landraces according to the province and the latitude from which they were collected. It also differed between subpopulations, being high in *indica 2* and *tropical japonica* and low in *indica 1* and *aus*. Drought scores suggest that *indica 2* is more drought resistant than the other groups. Correlations indicate that those landraces with high herbicide scores are more drought resistant in the vegetative stage. The landrace Niyan Wee, whose name in Sinhalese means “drought rice” belongs to the *indica 2* subgroup, has high herbicide scores and deep roots.

**Conclusions:**

Niyan Wee and other cultivars within the *indica 2* subgroup should be a valuable source of breeding for drought resistance at least partly because of their superior root traits, not normally associated with the *indica* rice cultivars.

**Electronic supplementary material:**

The online version of this article (doi:10.1186/s12284-016-0092-7) contains supplementary material, which is available to authorized users.

## Background

The rapid advance in genotyping technology including sequencing has led to an increased ability to accurately assess genetic variation across biological disciplines. In rice, the most important crop plant in terms of human consumption, this is exemplified by the development of SNP genotyping platforms (Zhao et al. 2011), low depth sequencing for genome wide association mapping (Huang et al. 2010) and now deeper sequencing of large collections (3K RGP 2014). The relative ease of genotyping promises to unlock some of the hitherto inaccessible diversity contained within local germplasm of our major crops (McCouch 2013).

In order to match genotype to phenotype, there has been a push to develop high throughput platforms that can provide relevant data on traits allowing genetic mapping to advance faster and more accurately. Araus and Cairns (2014) have reviewed advances in high throughput field-based phenotyping and highlight as an example “Shovelomics” for root traits where root numbers and their angle of growth can be assessed for a very large number of plants (Trachsel et al. 2011). One very simple root phenotyping method has recently been described where a herbicide is buried in a box of soil and the speed of symptom development related to rooting depth (Al-Shugeairy et al. 2014).

Rice is the staple food in Sri Lanka, where it is cultivated as a wetland crop in all the districts of the island and occupies 34 % (0.77/million ha) of the total cultivated area (De Silva and Yamao 2009). It provides 45 % of the calories and 40 % of the protein for the population (De Silva and Yamao 2009). One-third of Sri Lanka’s rice area are in rainfed lowlands or uplands, which are frequently subjected to water deficit of varying intensities and durations causing significant yield losses (De Costa 1998). During the Yala growing season (March to September: 310, 000 ha) a huge part of the island is drought-prone (De Costa 1998). According to the Climate Risk & Adaptation Country Profile of Sri Lanka 2011, (GFDRR 2011) the frequency of drought increased from 1974 to 2004.

Sri Lanka has more than 3,000 landraces of rice; the International Rice Research Institute have nearly 2,000 in their genebank while the Plant Genetic Resources Centre in Sri Lanka has about 3,000 (FAO 2010). Among these is a landrace called Niyan Wee which means “drought rice” in Sinhalese. The possibility exists that these landraces contain adaptation to specific climatic and edaphic conditions of Sri Lanka that might be usefully exploited for rice breeding in Sri Lanka and elsewhere. Furthermore, the possibility exists that the genetic diversity that is associated with this adaptation has not so far been significantly studied in projects assessing global variation in rice.

This paper describes a study on genetic diversity of 135 Sri Lankan accessions, almost all identified as landraces, including three accessions called Niyan Wee, with a local geographic focus using a marker system that allows comparison with global diversity panels. At the same time it assesses rooting depth using a new high throughput method that sheds light on potential adaptation mechanisms for drought that should prove useful in breeding programmes.

## Results

### Population structure and relation to drought scores

Based on analysis using STRUCTURE, it is most likely that the 129 Sri Lankan accessions analysed with 326 SNPs fall into six subpopulations since with K = 6 the Delta K value was over 16 while with K = 3–5 and 7–9 it was less than 2 (Additional file 1: Figure S1) (note 6 accessions were excluded from STRUCTURE analysis). The allocation of the cultivars to the K = 6 subpopulations is presented in Table 1. A more complete dataset including subpopulation, herbicide scores and data from the IRGCIS website is presented in Additional file 2: Table S1 while all the SNP data is presented in Additional file 3: Table S2.

**Table 1:** Sri Lankan landraces used in this study with the subpopulation they are clusters in and herbicide score for deep rooting

Landrace Name	Sub-population	Herbicide score	Landrace Name	Sub-population	Herbicide score	Landrace Name	Sub-population	Herbicide score
AHAMBA	In 1	1.75	INGRISI WEE	In 3	1.87	PANDURU WEE	In 3	1.25
AKURAMBODA	In 1	1.25	KAHATA SAMBA1	In 3	1.25	PANNITI	Aus	2
AL WEE	Aus	0.75	KAHATA SAMBA2	In 3	1.75	PERIYA KARUPPAN	In 2	3.37
ALAGUSAMBA	Ad	1.75	KAHATAWEE	Ad	1.25	PERIYA SIVAPPU	In 2	2.5
ALAKU SAMBA	Ad	2.37	KAL VELLAI	In 2	4	PERIYAVELLAI	In 2	3
ALE WEE	In 1	1.87	KALU BALAWEE	Ad	2	PODI NIYAN WEE	In 2	2.75
ALEWEE	In 1	1.25	KALU GIRES	Ad	1.75	PODI WEE2	In 2	2.12
ANDIKULAN	Aus	0.5	KALUBALAWEE	Aus	3	PODIWEE1	TJ	5
ARNOLISWEE	Aus	0.25	KALUKUDA	In 4	2.37	POKKURU SAMBA	In 1	1.25
BADHIWEE	In 3	1.75	KALUNDAI	In 2	4	PUKURU SAMBA	In 1	2.25
BALA KAHARAMANA	In 4	2	KARUPPAN	In 2	3.37	PUSHPARAGA	Ad	2.25
BALAMAWEE	In 1	1.87	KARUPPU SEENADHI	In 4	2.75	RAMBUTTANWEE	In 3	2
BATA POLA EL	In 4	1.62	KATHARAMANA	In 3	1.83	RANGAMODAN	In 2	3.25
BATAPALA	In 1	1	KIRIBATHA	In 4	3.12	RANKIRI	In 1	2.75
BATAPOLAWEE	Ad	1.5	KIRINARAN	Ad	2	RATA THAVALU	In 4	2.12
BATHIRATAWEE	In 3	2.12	KOLANATHIWEE	In 3	1.37	RATATHAWALU	Ad	1.75
BATUDELLA	TJ	3.62	KOTHTHAMALLI SAMBA	In 1	1	RATAWEE1	In 1	0.5
BG12-1	In 1	1.12	KOTTAMALLI	In 3	1.62	RATAWEE2	Ad	2
BW100	In 1	1.25	KURUBALAWEE	Aus	1.12	RATH RANPODIWEE	Ad	1.87
BW288-2	In 1	0.5	KURULUWEE	Aus	1	RATHKARA	In 3	1.87
CHITHIRA KALI	In 1	2.25	KURUWEE	In 1	2.37	RATHU HEENATI	In 1	2.62
DAMPOTAWEE	In 4	2.37	LUMBINI1	In 3	1	RATHU WEE1	In 3	3
DIK WEE2	In 1	1.62	LUMBINI2	In 4	2.12	RATHUWEE2	In 3	2
DIKWEE1	In 1	2.5	MA GODA AL	In 1	2.17	RUWANRATHRAN	In 3	1
DURU WEE2	Aus	1	MADAEL	Ad	3	SARALI	In 2	3.5
DURUWEE1	Ad	1.87	MADAKALAPUWEE	Aus	3.5	SEDUKKAN SAMBA	In 1	1.5
GALPAWEE	Ad	2.37	MADOLUWA	In 3	1.75	SINNAVELLAI	In 2	4.33
GAMBADAWEE	In 4	0.75	MADURU SAMBA	In 4	1.75	SUDU HONDARAWALA	In 1	3.37
GAMBODA	In 4	2.87	MAHABATHAN	Ad	2.25	SUDU MADA THAVALU	In 4	1.87
GODAWEL	TJ	1	MAHAGODA AL	In 1	2.62	SUDU MURUNGA	In 1	1
GONABARU	TJ	1.5	MAHARATAWEE	Ad	1.75	SUDUBALAWEE	In 4	2.25
HAL AL1	In 4	2.37	MALWARIYA	TJ	2.75	SUDURU SAMBA	Ad	2.37
HAL AL2	In 4	1.75	MATARAWEE	In 3	1	SULAI	In 1	2
HAL ELIYA	In 4	1.87	MATHOLUWA	In 3	2.25	TAIWAN	In 1	1.25
HAM-ALE-WEE	In 1	1	MENIKWEE	In 4	2.5	THAHANALA	Aus	2.83
HEEN GODA WEE	TJ	3	MODDAI KARUPPAN	In 2	3	THATUWEL	TJ	3.25
HEENATI1	Aus	1.25	MOLLIGODA	In 3	2.37	THAVALU1	Ad	2.12
HEENATI2	Aus	3	MOOTHUKIRIEL	In 4	2.25	THAVALU2	In 4	1.87
HEENDIKWEE1	Ad	2	MOROGALLAWEE	Ad	2.12	TISSA WEE	In 3	1.75
HEENDIKWEE2	In 3	1	MUDUKIRIEL	In 1	2.25	VELLAI	In 2	3.62
HEENDIKWEE3	In 1	1.75	MUTHU SAMBA	In 1	2.5	VELLAI ILLANKALI	In 1	1.25
HONDARAWALA1	Ad	2.12	MUTHUSAMBA	In 1	3.25	WANNI DAHANALA	In 4	2
HONDARAWALA2	In 4	2.5	NIYAN WEE1	In 2	4	WEDA HEENATI1	In 3	2.62
HORANA MAWEE	In 4	2.5	NIYAN WEE2	In 2	4	WEDA HEENATI2	Aus	1.87
ILLUPAIPU SAMBA	Ad	1.5	NIYAN WEE3	In 2	3	YAKADAWEE	In 1	2.25

Further information including accession numbers, drought scores, maturity data and geography of their collection is provided in Additional file 2: Table S1

**Fig. 1:**
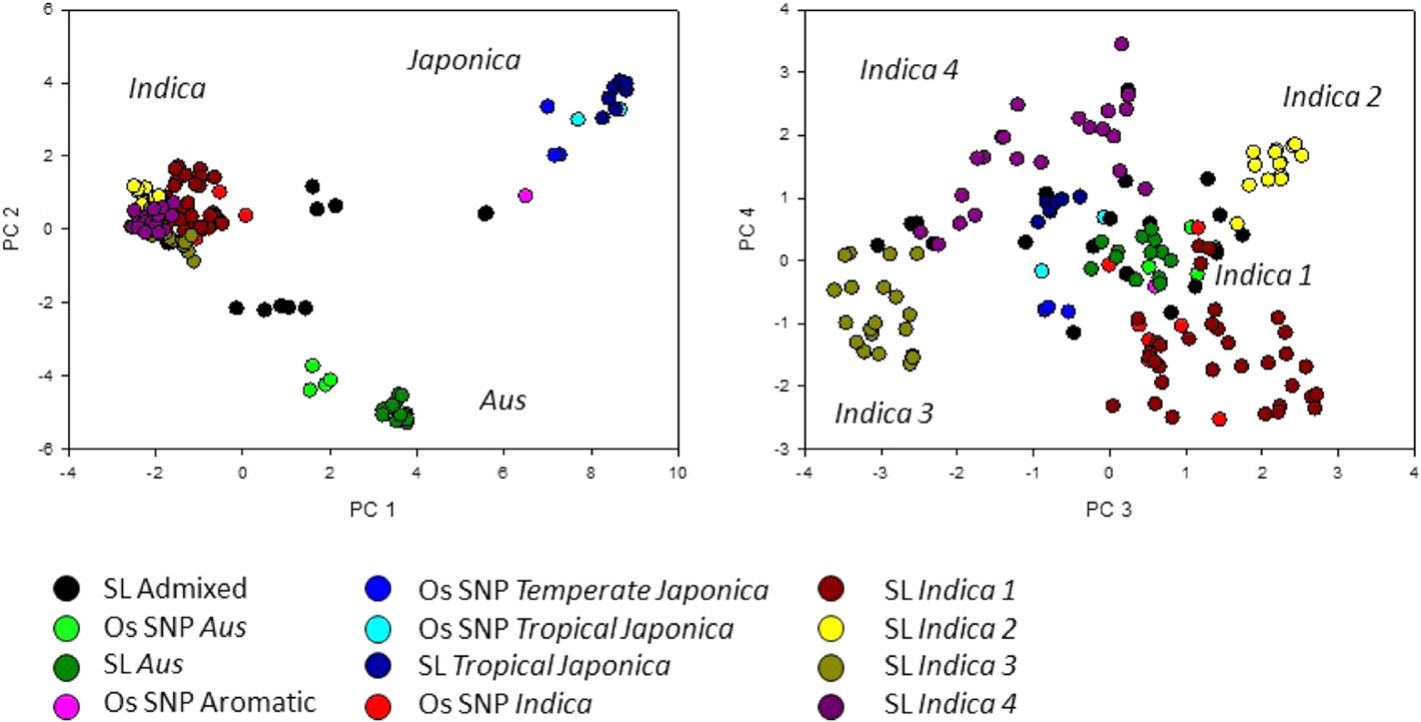
Plots of the top four axis of principle components analysis where accessions have been colour coded to allow comparisons. SL means Sri Lankan landrace, Os SNP means OryzaSNP accession. Loadings are PCA 1 = 22.8 %, PCA 2 = 9.2 %, PCA 3 = 7.2 % and PCA 4 = 4.2 %

A principal components analysis with 129 Sri Lankan accessions plus 18 accessions of the OryzaSNP is given in Fig. 1. It can be used to visualise the groupings identified by STRUCTURE. PC axis 1 (22.8 % loading) separates *Indica* (which includes OryzaSNP accession Aswina, Zhenshan 97, IR 64, Sanhuangzhan 2, Swarna and Minghui 63) from *Japonica* (which include OryzaSNP *tropical japonica* accessions Azucena and Moroberekan and *temperate japonica* M202, Nipponbare and Tainung 67) and PC axis 2 (9.2 %) separates *indica* from *aus* (which includes OryzaSNP accessions FR13A, Rayada, Dular and N22) (Fig. 1a). A combination of PC axis 3 (7.2 %) and PC axis 4 (4.2 %) clearly separate the *indica* subpopulations (Fig. 1b). This figure also clearly shows the relationship between the Sri Lankan accessions and the 18 OryzaSNP accession where the latter have no representation in *indica 2, 3* or *4*.

**Fig. 2:**
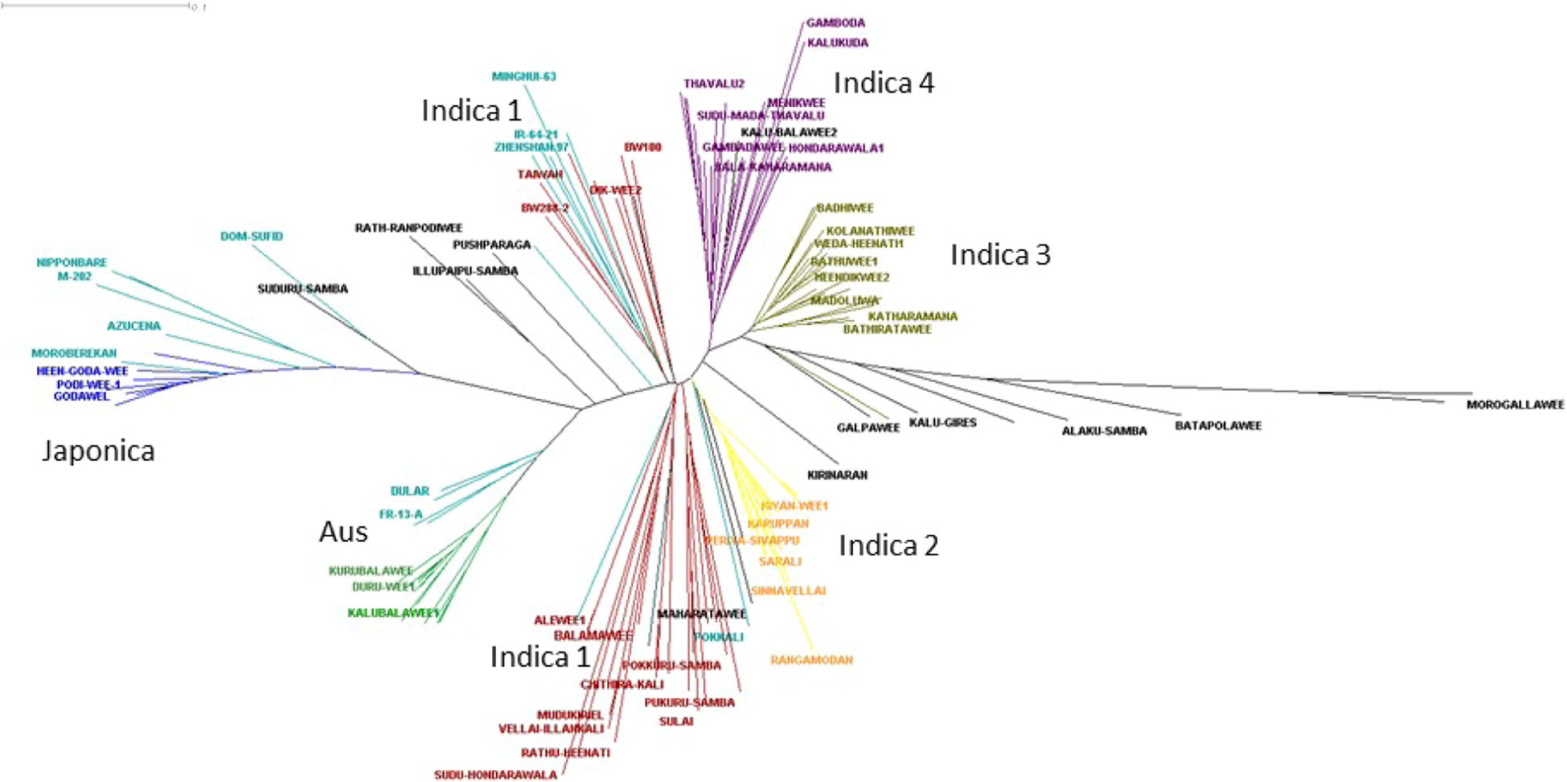
Unrooted dendrogram of 135 Sri Lankan accessions and 18 OryzaSNP accession based on 326 SNP markers using colour codes of Fig. 1. This tree is available as a nexml file to open in Dendroscope as Additional file 4: Online Resource 1

A dendrogram of the 135 accessions with the 18 OryzaSNP accessions is given in Fig. 2 where each subpopulation is colour coded allowing the general pattern observed in the PCA to also be observed (note this tree is not bootstrapped so inferences must be treated with caution). A “nexml” format file of the NJ tree shown in Fig. 2 that can be visualised using Dendroscope is provided as Additional file 4: Online Resource 1. The OryzaSNP accessions are identifiable as cyan in the tree. OryzaSNP *aus* accession FR13A, Rayada, Dular and N22 clustered with, but were distinct from, 13 Sri Lankan accessions including Alnoliswee, while OryzaSNP *tropical japonica* accession Moroberekan and Azucena cluster within seven Sri Lankan accessions including Podiwee-1. *Temperate japonica* OryzaSNP accessions M202, Nipponbare and Tainung 67 cluster with, but are distinct from these *tropical japonica.* All the rest of the Sri Lankan accessions are considered to be *indica* because known *indica* OryzaSNP accessions Aswina, Zhenshan 97, IR 64, Sanhuangzhan 2, Swarna and Minghui 63 cluster with one group (called here *indica 1*) which contains 33 Sri Lankan accession. This *indica* 1 group includes Sri Lankan accessions BG12-1, BW100 and BW288-2, the only three of the accessions used here identified as breeding lines, not landraces. Three other groups recognised by STRUCTURE contain no representatives from the OryzaSNP set but appear to be *indica* since they are close to the *indica 1* group. The OryzaSNP cultivar Pokkali, a salt tolerant *indica* from India clusters close to the group called here *indica 2* which contains 16 Sri Lankan landraces including all three accessions named Niyan Wee. *Indica 3* contains 22 Sri Lankan landraces while *indica 4* contains 22. This leaves 22 Sri Lankan landraces considered as admix between these six subpopulations. This includes three landraces Suduru-Samba, Thavalu 1 and Durwee 1 which clustered with the aromatic OryzaSNP accession Dom Sufid suggesting these three might be aromatic.

Examining the dendrogram produced when the Sri Lankan landraces were combined with the RDP1 (for 267 SNP markers) suggests that the *indica 2*, *3* and *4* subpopulations are not represented in RDP1 while *indica 1* is (data not shown but nexml file supplied as Additional file 5: Online Resource 2). For example, Sri Lankan accessions Alewee 1, Alewee 2 and Ham-Ale-Wee (*indica 1*) cluster near RDP1 accession JM70, an *indica* from Mali, and Ratawee 1 (*indica* 1) clusters with RDP1 accession Jaya, an *indica* from India and quite close to the improved variety IR 8. A demonstration that this analysis is valid is provided by the fact that the *aus* landrace Kalubalawee used here clusters with the Sri Lankan *aus* RDP1 accession Kalubala vee. Another Sri Lankan accession in RDP1 (Rathuwee, an *indica*) clusters near the breeding line used here BW288_2 which is from *indica* 1 and not with the Sri Lankan landrace used here in *indica 3* that is also called Rathuwee. The other Sri Lankan accessions in RDP1 (LD 24, an *indica*) does not cluster with the Sri Lankan landraces used here.

**Fig. 3:**
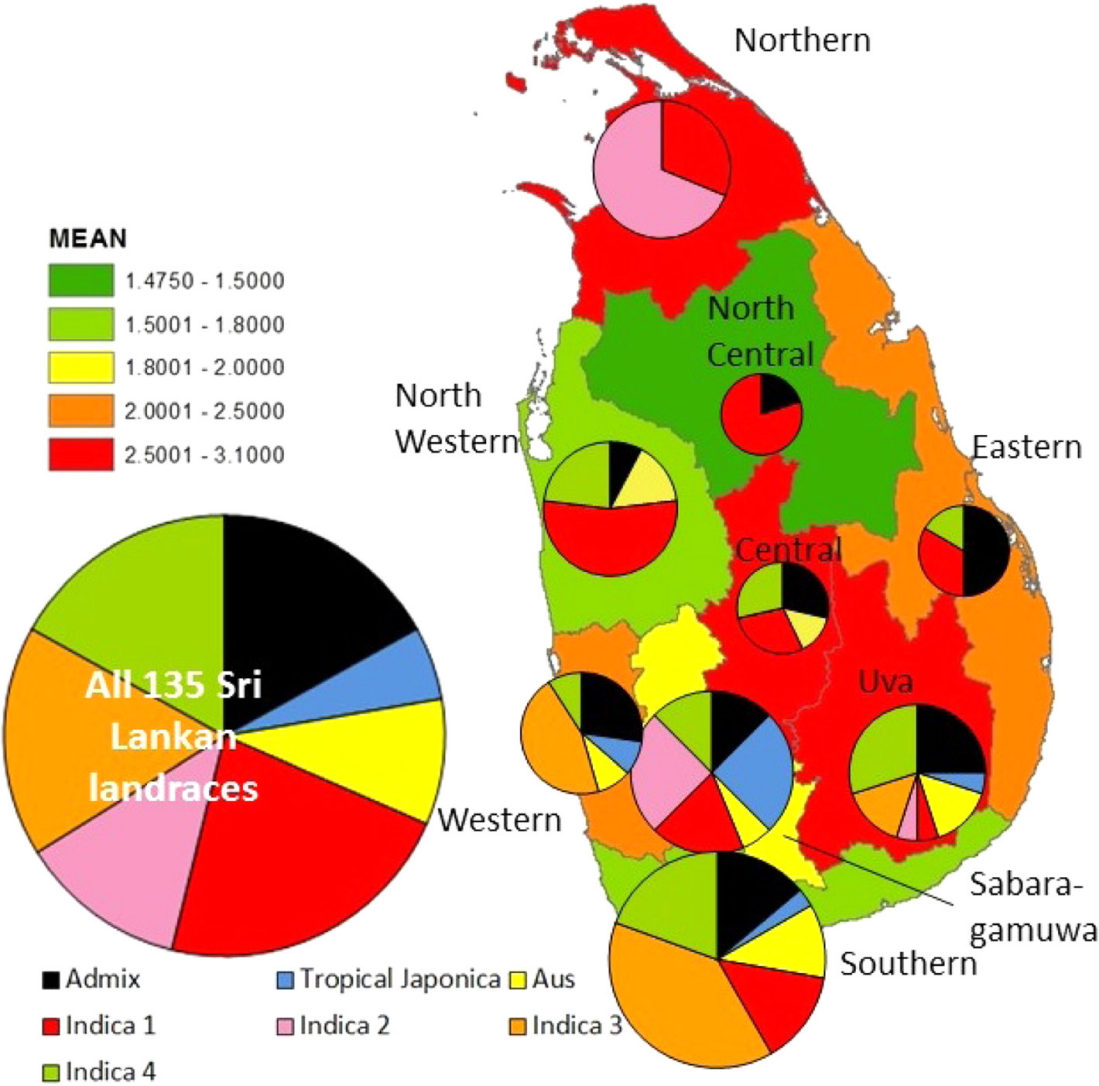
Provinces of Sri Lanka colour coded by herbicide score with pie graphs indicating group of landraces by province (area proportional to number of accessions)

Examining the distribution of the landraces of different subpopulations identified by the province of Sri Lanka from which they came shows they are not uniformly distributed (Fig. 3). *Tropical japonica* and *aus* landraces used in this study come from the south or the south and west of the county respectively while *indica 1* s are quite evenly spread. Most of the *indica 2* s come from the North but Niyan Wee is from Uva (South and East of the centre) and one comes from Sabaragamuwa (South and West of the centre). The *indica 3* subpopulation appears to come from the South and Southwest of the country while *indica 4* are present throughout the country except the two most northerly provinces.

**Fig. 4:**
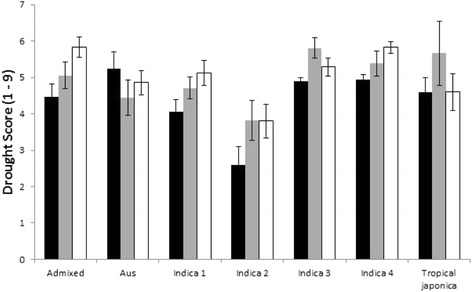
Mean drought scores obtained from IRGIS database for Sri Lankan landraces grouped according to identified subpopulation. Black is drought score 3, grey is 4 and open is 5. In all cases, low numbers represent high drought resistance. Bar is standard error

One way analysis of variance (ANOVA) of date of maturity using the identified subpopulations was significant (*P* = 0.001) and when the outlying *indica 1* landrace Koththamalli Samba (224 days) was removed (160 days is the next highest maturity date) it was clear from a Tukey’s test that *indica 2* are longer duration (146 days) compare to the other subpopulations all of around 130 days. ANOVA was also conducted on the drought score values. There were significant differences between subpopulations for drought score 1 (seedling vigour; *P* = 0.05), 3 (recovery after 2^nd^ stress; *P* < 0.001), 4 (drought resistance at early vegetative stage; *P* = 0.017) and 5 (drought resistance at late vegetative stage; *P* = 0.001). Most striking were for drought 3 where 30 % of variation was explained by subpopulation and a Tukey’s test revealed *indica 2* was more drought resistant than the other 5 subpopulations, but it is noteworthy that drought 4 and 5 also revealed *indica 2* are remarkable for possessing low scores indicative of high drought resistance (Fig. 4).

### Root traits

Symptoms of herbicide-induced plant injury were first detected on Podiwee and Niyan Wee 2 on day 20 and by day 21 genotype explained 36 % of the variation in herbicide score (from one way ANOVA). The proportion of variation explained by genotype slowly increased to peak at 49 % on day 31, after which it decline very slowly as more and more plants reached a score of 5. All herbicide scores (at different days) correlated very strongly with each other, with herbicide scores on day 31 having *r* = 0.935 and *r* = 0.872 for correlations with day 37 and day 43 respectively and day 37 and day 43 with *r* = 0.920 (see Additional file 6: Table S3 for more correlations between herbicide scores on different dates). A histogram of mean herbicide scores at day 43 is presented in Additional file 7: Figure S2 when 43 % of variation was explained by cultivar. The herbicide score at day 43 was chosen as the most appropriate to examine relationships with genetic diversity and plant phenotype traits as it was roughly normally distributed while earlier scores are skewed by large numbers of landraces with scores of zero. As expected, check cultivar Black Gora had a high herbicide score throughout (over 3 by day 43) but was surpassed by Podiwee 1, Niyan Wee 1, Niyan Wee 2 and Sinnavellai. Poor-rooted check varieties Bala and IR 64 showed very little herbicide symptoms (below 1 by day 43). Some landraces showed equally low herbicide scores such as Arnoliswee and Ratawee 1. Herbicide scores correlated with drought scores 3, 4 and 5 only, and were strongest for drought 5 (late vegetative drought resistance) where herbicide score at day 31 and 43 gave correlation coefficients of *r* = −0.335 and −0.350 respectively (*p* = 0.001) indicating that a high herbicide score is associated with a low drought score (high drought resistance).

**Fig. 5:**
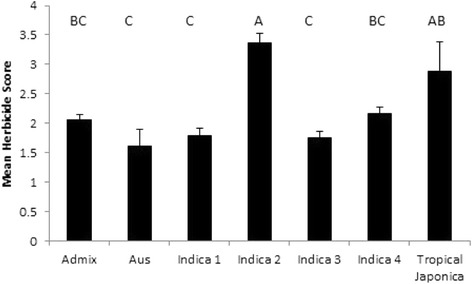
Mean herbicide score at day 43 for Sri Lankan landraces grouped according to identified subpopulation. Bar is standard error. Columns sharing the same letter are not different

Herbicide score was significantly associated with subpopulation and a Tukey’s test on herbicide score at day 43 revealed three groups, where *indica 2* had high scores, and *indica 1*, *3* and *aus* had low ones (*P* < 0.001) (Fig. 5). The herbicide score was also very strongly associated with province and district, with these factors explaining a remarkable 28 and 35 % of the variation in one way ANOVA. The mean herbicide score according to province is presented on the map of Sri Lanka in Fig. 3 which shows that high herbicide scores are found in Northern, Central and Uva provinces. Herbicide score is also highly significantly related to the latitude from which the landraces were collected (*P* < 0.001; *R* ^2^ = 20 %) where those from the North (latitude 9°) had a higher score than others (from latitudes 5–8°) according to a Tukey’s test.

**Fig. 6:**
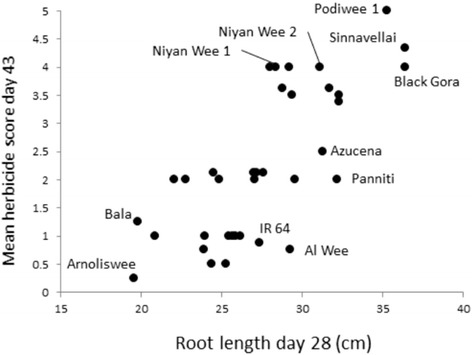
Plot of hydroponic root length at day 28 vs. mean herbicide score at day 43

Root and shoot traits measured on 36 hydroponically-grown rice genotypes are given in Additional file 8: Table S4. All traits were highly discriminatory between genotypes, (R^2^ ranged from around 33 % for root/shoot dry weight ratio, root thickness and root mass to 50–60 % for maximum root and shoot length). A Tukey’s test comparing the three chosen groups of Sri Lankan accessions (those with high, average and low herbicide scores) showed that root length and root weight distinguished the high herbicide accession from the rest, but root thickness and % root mass (mass of root over mass of plant) did not. Most traits measured in hydroponics correlated with herbicide score, but the correlations were strongest with root traits (see Additional file 9: Table S6 for complete correlation data). The strongest correlation of all was with maximum root length at day 28 where *r* = 0.750 (Fig. 6) whereas the strongest correlation for shoot traits was with shoot length at day 35 (*r* = 0.586). Figure 6 shows that check varieties behaved as expected (as shown in Shrestha et al. 2014), with Black Gora very long rooted, Azucena reasonably long rooted, IR 64 reasonable short rooted and Bala very short rooted. There were Sri Lankan landraces across the spectrum of these check varieties including some very long and very short rooted.

**Fig. 7:**
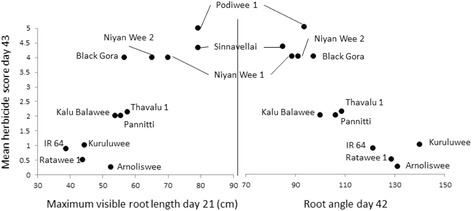
Scatter plots of rhizotron root length (left) and root angle (right) against mean herbicide score

All of the 27 traits measured on the 12 genotypes grown in rhizotrons (presented in Additional file 10: Table S5) were highly discriminatory between genotypes (*P* < 0.005) with R^2^ values from one way ANOVA ranging from 27 % (number of roots past 50 cm depth at 35 days) to 83 % (shoot length at 42 days). For maximum visible root length at day 35 and day 42 R^2^ was 62 and 60 % respectively. Almost all traits also correlated with herbicide score at day 43 (see Additional file 11: Table S7 for complete correlation data) with the two strongest being root length at day 21 (*r* = 0.881) and root angle at 42 days (*r* = −0.901). These remarkable correlations are presented in Fig. 7. The lowest correlation was with shoot length at day 14 (*r* = 0.603).

## Discussion

### Sri Lankan landraces are genetically distinct

The rapid reduction in the cost of genotyping should mean that for world crops a better appreciation of local diversity within the context of the worldwide genetic variation of the species will become available. As yet, however, this process is only just beginning and is very limited for Sri Lankan rice. The Rice Diversity Panel 1 which has been genotyped with 44,000 SNPs (Zhao et al. 2011) has only three Sri Lankan accession, a recent expansion of that set to 1571 accessions of the Rice Diversity Panel 2 (RDP2) with 700,000 markers has 48 (McCouch et al. 2016) while the recent sequencing of 3,000 rice accessions (3K RGP 2014) includes only 47 Sri Lankan accessions. Of the 48 Sri Lankan accessions in RDP2, only four have the same name as used here and none have the same IRGC id (that is KURUWEE, MADAEL, RATHUWEE and WEDA HEENATI). Of the 47 accessions of the 3 K rice genomes project only four are the same as used here (8944 ALAGUSAMBA, 8901 MATHOLUWAA, 15485 MODDAI KARUPPAN and 11938 PODIWEE) while three have the same name but different accession numbers to this study (HEENDIKWEE, HONDARAWALA and KURULUWEE). A study using AFLPs to examine specifically Sri Lankan rice assessed 74 accessions plus six wild rices (Rajkumar et al. 2011), applying 772 molecular markers to characterise local diversity but since the marker system does not transfer to other studies, placing those results into the wider context of global diversity is difficult. In the study reported here, by using SNP markers that have been characterised on the OryzaSNP set (McNally et al. 2009) and most of which have been tested on the RDP1, the global context is more easily evaluated.

Structure analysis reveals six subpopulations within 129 of the accessions, which by comparative analysis with 18 OryzaSNP cultivars can be identified as *aus*, *tropical japonica* and four which appear to be *indica*. This grouping is clearly demonstrated in PCA plots of Fig. 1. This grouping is not reflected in the two large clusters of Sri Lankan accessions identified by Rajkumar et al. (2011); 14 of the accessions have common names revealing that their “cluster 2” contains 10 landrace used here from *indica 1*, *3*, *4*, *tropical japonica* and admix while their “cluster 3” has four landraces used here from *indica 1* and *2*, *aus* and admix. The splitting of *indica*s into distinct subpopulations is not common in rice diversity studies but has been reported in China (Huang et al. 2010) where evidence showed latitude played a role in differentiating three *indica* subpopulations. Analysis of the 3,000 sequenced rice genomes presented in Alexandrov et al*.* (2015) also suggests the presence of three *indica* subpopulations but the details of the structure analysis method are lacking and it is not possible to reliably compare results with those presented here.

While the four *indica* subpopulations reported here were roughly equally represented in the landraces as a whole, and the *indica 1* subpopulation was represented in almost all of Sri Lanka’s 9 provinces (not Western Province), the *indica 2* and *3* subpopulations were found in only three provinces while the *indica 4* subpopulation was absent in the two most northerly provinces (Fig. 3). Clearly, therefore, these subpopulations of *indica* do not appear to be randomly distributed throughout Sri Lanka.

What seems to be relatively clear is that the *indica 1* subpopulation is typical of *indica* present in global collections but this does not appear to be true for the other three. This is suggested because the OryzaSNP *indicas* cluster in *indica 1* (Figs. 1b and 2) and when the RDP1 data of 267 SNPs are analysed with the Sri Lankan cultivars, *indica 1* landraces cluster with RDP1 *indica* but *indica 2*, *3* and *4* landraces do not. This implies that the cultivars from *indica 2*, *3* and *4* represent germplasm and genes that may have so far been poorly studied by the global rice community.

### The herbicide screen reveals differences in root traits

The herbicide screening system which has been recently developed to screen rice (Al-Shugeairy et al. 2014) proved highly discriminatory when used on the 135 Sri Lankan accession. This revealed some landraces having higher herbicide scores than Black Gora, the deep rooted check. When developing the high throughput method it was observed that there are differences in sensitivity of accessions to the herbicide (Al-Shugeairy et al. 2014). The authors commented that this was small compared to difference observed in the timing of symptoms when the herbicide is buried, but they nevertheless cautioned that the screen is a positive screen for deep roots since a plant that develops symptoms rapidly must be deep rooted while a plant that develops symptoms slowly or not at all might be shallow rooted or be insensitive to the herbicide. For this reason, the reliability of the herbicide screen to deduce that accessions differ in rooting depth was confirmed by a hydroponic screen on 36 genotypes and a rhizotron screen on 12 where Sri Lankan landraces from high, low and average herbicide scores plus check accessions were used. In both cases very high correlations were revealed between herbicide scores and root growth measured directly giving great confidence that these do reflect genuine differences in rooting behaviour. The fact that all traits measured in these root screens, including shoot traits, correlate with herbicide score indicates that an underlying driving factor for herbicide score is seedling vigour. However, the fact that the most strongly associated traits are root traits, not shoot traits and that in rhizotrons root angle is strongly correlated to herbicide score emphasises that the development of symptoms is most strongly related to the direction and length of root growth rather than any other trait. This confirms the observations in Al-Shugeairy et al. (2014) and should strengthen confidence about using this method for assessing large numbers of genotypes so long as the caveats detailed in that publication are understood. Confidence in this method should also be taken from the fact that the herbicide score is correlated negatively with drought scores 3, 4 and 5.

### Diversity in Sri Lankan rice is associated with rooting depth and drought resistance

The herbicide score discriminates between subpopulations of Sri Lankan landraces such that *indica 2* is high and *indica 1*, *3* and *aus* are low. In general it is reported that rooting depth of rice accessions differs between subpopulations. For example Shrestha et al. (2014) report that rhizotron screens rank subgroups in the order *aus* > *tropical japonica* > *indica* ≈ *temperate japonica* for maximum root length which was in agreement with an earlier report of Lafitte et al. (2001). This order has been confirmed testing the RDP1 with the herbicide screen (unpublished results) where *aus* has the highest average scores, followed by *tropical japonica* with *indica* and *temperate japonica* both similarly low. In this context, two surprising observations are made in the current study. First is that the Sri Lankan *aus* are low, and may reflect the fact that these landraces are somewhat genetically distinct from global *aus* cultivars. That this is the case is suggested by the observation that they do not cluster exactly with the OryzaSNP *aus* accessions (Figs. 1a and 2) and examining the dendrogram produced when the Sri Lanka accessions are combined with the RDP1 data (Additional file 5: Online resource 2), when they form a distinct cluster within the *aus* which only has RDP1 cultivars Kalubala Vee (a Sri Lankan aus) and Khao Pahk Maw (an aus from Thailand). The second, highly noteworthy observation is that the *indica 2* subpopulation has high herbicide scores (Fig. 5) which when considered in the context of the genetic data discussed above suggests that *indica 2* represents a distinct type of *indica* that has not previously been studied and which is deep rooted. This group includes Sinnavellai that was the cultivar with the 2^nd^ longest roots in rhizotrons (Fig. 7) and all three cultivars called Niyan Wee, two of which were tested in the rhizotrons and had root traits surpassing the deep rooted check Black Gora (note they did not surpass Black Gora in hydroponics while Sinnavellai matched it).

The subpopulations are also differentiated in the distribution of their drought scores such that the *indica 2* subpopulation appears to be more drought resistant, at least in terms of drought in the vegetative stage and recovery from stress (from drought score 3, 4 and 5) (Fig. 4). This emphasises the unique and potentially valuable nature of the landraces in *indica 2* and, taken together with the paragraph above implies that these cultivars are have a drought resistance that is linked to root growth.

## Conclusions

The study characterises 135 Sri Lankan accession, almost all landraces, and suggests the existence of genetic diversity within *indica* that has not been studied until now. It further shows that *indica 2* group has deep roots (unlike most *indica*) and high drought resistance. Since Niyan Wee is in this group, and its name means “drought rice” it seems highly plausible that accessions with this name are drought resistant and they certainly have deep roots when tested in herbicide screening and rhizotrons. These landraces and their partners in this subpopulation should be more thoroughly studied in order to determine the genetic and physiological background of their drought resistance and rooting traits.

## Methods

### Plant material

A total of 135 Sri Lankan rice accessions were supplied by the International Rice Research Institute (IRRI) chosen based on information on the International Rice Genebank Collection Information System database (http://www.irgcis.irri.org:81/grc/IRGCISHome.html). Only those identified on IRGCIS as landraces were considered except three breeding lines BG12-1, BW100 and BW288-2. From more than 2,000 Sri Lankan accessions listed, the landraces were further selected to represent most of the geo-climatic regions of the wet zone and dry zone of the country. The geographical location of each landrace including the province and district with longitude and latitude were obtained by searching detailed germplasm information of IRGCIS database for collecting location data (http://www.irgcis.irri.org:81/grc/SearchData.htm). Landraces were also chosen if there was available drought scores in the IRGCIS database (searching detailed germplasm information for reaction to physio-chemical stress) to ensure a range of scores. This data gives nine categories of drought score, Drought 1–9 but for only the first 5 are there sufficient data points to be valuable (>41). That is, in order; 1 = Seedling vigour (data for 117 accessions); 2 = Rate of recovery after the 1st stress (data for 107 accessions); 3 = Rate of recovery after the 2nd stress (data for 90 accessions); 4 = Drought resistance score at early vegetative stage (50–60 days) (data for 110 accessions); 5 = Drought resistance score at late vegetative stage (80–100 days) (data for 88 accessions). In all cases, drought scores are given on a scale of 1 to 9 where a high value indicates highly sensitive to drought.

A list of the accessions is given in Table 1 and full detail of the IRGC accessions is provided in Additional file 2: Table S1. It includes some accessions which share the same name. Where this occurs, they have been given a number to distinguish them given by ascending order of accession number. Thus there are three accessions called Niyan Wee, which are named here Niyan Wee 1, 2 and 3 for accession numbers 66525, 67646 and 67647 respectively.

In addition to the Sri Lankan material, four check cultivars Azucena (*tropical japonica*), Bala (*indica*), Black Gora (*aus*) and IR 64 (*indica*) were used for root screening, using seeds from plants grown in Aberdeen but originally supplied from IRRI.

### Genotyping using a 384 SNP array and genetic analysis

DNA was extracted from fresh leaves using the Invitrogen ChargeSwitch gDNA plant kit (Invitrogen, Paisley, UK) using the manufacturer’s instructions. The DNA was analysed on a custom designed 384 SNP Illumina GoldenGare array using the Illumina Beadxpress platform at the Molecular Marker Laboratory at the James Hutton Institute, Invergowrie, Dundee, DD2 5DA, Scotland, UK. The design of the 384 SNP array is described in Travis et al. (2015) but briefly it use Veracodes listed on the Rice Diversity web site (http://www.ricediversity.org/data/index.cfm) selected to include evenly-spaced SNPs with a mixture picked from the RiceOPA1.0 (quality control), RiceOPA2.1 (*Indica/Indica*), RiceOPA3.1 and RiceOPA7.0 (*Indica/Japonica*) and RiceOPA4.0 (*Japonica/Japonica*) SNP sets.

From the 384 SNPs, data of 58 markers were removed because of recognisable variation between plates of 96 samples. All SNP data generated from 326 markers (given in Additional file 3: Table S2) were combined with data for the same markers for 18 accessions from diverse set of 20 rice cultivars and landraces in the OryzaSNP panel (McNally et al. 2009) in order to use them as check accessions of known subpopulation origin in identifying different groups in population structure analysis.

Cluster analysis of SNP data were conducted using TASSEL (Trait Analysis by aSSociation, Evolution and Linkage) Version 4 (Bradbury et al. 2007). This software produced a data matrix showing relative closeness of accessions based on SNP data. Neighbour joining clustering method was used in this analysis and TASSEL produced a dendrogram (tree plot) which was visualized in detail in software Dendroscope (Huson and Scornavacca 2012). For 267 of the SNPs used here, data for the 372 accessions of the Rice Diversity Panel 1 (Zhao et al. 2011) are available from the Rice Diversity web site. TASSEL was used to produce a dendrogram of these accessions with the 135 Sri Lankan landraces. Both of the trees produced this way are available as online resources that can be opened in Dendroscope.

A total of six landraces (Alaku-samba, Batapolawee, Heendikwee-1, Kahatawee, Madael and Morogallawee) which produced unusually long branches in the dendrogram were removed from the list before further analysis to determine the number of populations within the Sri Lankan accessions. Population structure in 129 Sri Lankan accessions (without the OryzaSNP cultivars) was analysed in STRUCTURE Version 2.3 (Pritchard et al. 2000) software by implementing a model - based clustering method for inferring population structure. The model consisted of 10000 burn-in period and 50000 MCMC replicates. The program was run with number of populations assumed (K) from 1 to 10 each with 10 replicates. The true number of subpopulations (K) was determined by plotting a graph of K versus Delta K using mean log likelihood for each K (Ln P (D)) as described in the tutorial of the STRUCTURE software (Additional file 1: Figure S1). The quantitative genetics program TASSEL (Bradbury et al. 2007) was used to perform Principal Component Analysis (PCA) of the SNP data including the OryzaSNP set of 18 accessions, after filtering and imputation of missing SNPs using default parameters.

### Assessing rooting depth using buried herbicide

The buried herbicide method for assessing rooting depth in rice has been described in full (Al-Shugeairy et al. 2014). Briefly, a box of dimensions 386 cm long x 81.5 cm wide and 40 cm deep was made in a greenhouse using plywood covered in plastic sheet. In the bottom, a 5 cm deep layer of sieved loam topsoil (Rolawn, UK) was watered with 11.75 l of Yoshida’s nutrient solution (Yoshida et al. 1976). This was covered in filter paper soaked in TRIK (active ingredients Diuron (46.4 %), Aminotriazole (26.6 %) 2,4-D (11.2 %) herbicide (no longer available)) at a dose of 100 mg of herbicide per plant. Two soil moisture meters (Theta Probe, Delta-T, Cambridge) were placed above the filter paper and a depth of 15 cm more soil added before a further two moisture probes were added. A further 15 cm of soil was added (meaning the herbicide layer was 30 cm below the soil surface) and the whole box watered with 65 l of nutrient solution. The 135 Sri Lankan accessions plus five check cultivars (Azucena, Bala, Black Gora, IR 64 and Nipponbare) were sown 5 cm apart in four replicate blocks in a randomised complete block design on the 19^th^ July 2010. The greenhouse conditions maintained a minimum temperature of 25 °C. While there was no supplemental lighting, at this time of year in Aberdeen (57° latitude) the day length is very long (going from 17.75 h to 15.5 h during experiment) so the plants received a relatively high amount of light (but it was not monitored). The box was watered to maintain a volumetric water content between 24 and 25 % with tap water. After 19 days plants started to show symptoms of herbicide-induced damage and were scored every day using a visual “Herbicide Score” of 0–5 where 0 is no symptoms, 1 is given when 5–15 % of leaf area shows visible symptoms (leaf yellowing), 2 when 15–50 % of leaf area shows visible symptoms, 3 when more than 50 % of the leaf area shows symptoms, 4 when 15–50 % of the leaf area is dead and 5 when more than 50 % of the leaf area is dead. Note this score has been shown to be essentially parametric by correlation with % affected leaf area score in subsequent screens (Al-Shugeairy et al. 2014).

### Assessing root traits using hydroponics

A total of 32 Sri Lankan landraces plus check cultivars Azucena, Bala, Black Gora and IR 64 were assessed in a hydroponic system previously described (Price et al. 1997, Shrestha et al. 2014). The Sri Lanka landraces were chosen to represent 11 with the highest herbicide scores, 11 with lowest scores and 10 with near average scores. Four completely randomised replicate boxes containing 38 L of nutrient were used. Seeds were washed in tap water, surface sterilised in 1 % sodium hyperchlorite then pre-germinated in Petri dishes at 37 °C for two days. Germinated seedlings were planted in plastic trays fitting in to covered plastic tubs with aerated Yoshida’s nutrient solution in a controlled environment growth room under a 12 h light regime with a light intensity of approximately 400 μmol m^−2^ s^−1^ PAR for 12 h per day with 25 °C at night and 28 °C in the day. The nutrient solution was replaced every week while maintaining a constant pH of 5.5 daily. The maximum root and shoot length of each plant were recorded weekly for 5 weeks. After 35 days the plants were harvested, roots and shoots were separated, dried and weighed. The root thickness of each plant was measured with a Leitz stereo dissecting microscope using 1 cm long pieces of three thick nodal (adventitious) roots sampled near the base of the plant which were kept in water at 4 °C for a maximum of one week.

### Assessing root traits using rhizotrons

A total of 12 genotypes were assessed for root traits using the rhizotron system that is described in detail in Price et al. (2012) which has been widely used to assess rice (Price et al. 2002; Shrestha et al. 2014). Four replicates were arranged in completely randomised blocks. The genotypes were the four highest, three lowest and three average herbicide scoring Sri Lankan landraces plus check cultivars Black Gora and IR 64. Briefly, the rhizotrons were made by taping together two sheets of 4 mm thick glass (1.2 x 0.3 m width x length) 15 mm apart with duct tape and filling the space with topsoil (as used in herbicide box). Approximately 7 kg of moist (20 % volumetric water content) soil were used to give a dry bulk density of about 1.1 kg l^−1^. The chambers had 5 mm diameter drainage holes at the bottom of each side. Before sowing, each chamber was saturated with 500 ml of Yoshida’s full strength nutrient solution at pH 5.5 (Yoshida et al. 1976). Two seeds were sown in each chamber on 20th July 2011. After seedling emergence, the seedlings were thinned to one. Plants were grown for 6 weeks in a greenhouse (minimum temperature 25 °C) under daylight conditions (no supplementary lighting). Each rhizotron was connected to a drip irrigation system and supplied with 250 ml of Yoshida’s nutrient solution three times a week for the first 3 weeks, and 5 times a week for the final three weeks. In the final week rhizotrons received 125 ml of water a day in addition to the 250 ml nutrient solution. Plants received their last watering on the evening of day 39 after sowing and received no more water until harvested on day 42.

On a weekly basis shoot growth was monitored as shoot length (length from the soil to the tip of the longest leaf) while the length of the longest visible root and the number of roots passed 25 cm at 21 days, 50 cm at 35 days, 75 cm at 42 days were recorded. On day 21 and 42 the angle of spread of the root system (the maximum angle between the least vertical main root axes on either side of the plants base) was measured with a protractor. The weight of each rhizotron was measured every day from day 39 until day 42 to assess water use in the final three days of the experiment. On the evening of day 39, a digital image was taken of each rhizotron using a 12 MPixel camera. On day 42 shoots were removed in a single day, dried at 80 °C for two days and subsequently weighed. Over a 1-week period, each chamber was opened. Short sections of three of the thickest nodal (adventitious) roots were removed from each root system at the base of the shoot, placed in water and stored at 4 °C, before being used to assess root thickness under a Leitz stereo dissecting microscope. The entire root system was divided into 3 sections, 0–40 cm (the top), 40–80 cm (middle) and 80–120 cm (bottom), washed, dried and subsequently weighed.

## Additional files

Additional file 1: Figure S1. Optimum K value of 6 revealed in the K vs delta K plot from STRUCTURE. (DOCX 24 kb)

Additional file 2: Table S1. Accessions, subpopulation, drought scores and mean herbicide score. (XLSX 49 kb)

Additional file 3: Table S2. Genotype of 326 SNPs for 135 Sri Lankan landraces plus 18 OryzaSNP accessions (the last 18 entries). (XLSX 198 kb)

Additional file 4: Online Resource 1 (NEXML tree of SL and OryzaSNP accessions 152 kb)

Additional file 5: Online Resource 2 (NEXML tree of SL and RDP1 accessions 127 kb)

Additional file 6: Table S3. Correlations between herbicide scores (HS) at different days. (DOCX 12 kb)

Additional file 7: Figure S2. Frequency distribution of mean herbicide score at 43 days for 135 Sri Lankan landraces. (DOCX 43 kb)

Additional file 8: Table S4. Mean of traits measured in hydroponics with summary test statistics for one way ANOVA at the bottom. (XLSX 16.2 kb)

Additional file 9: Table S6. Correlations between herbicide score day 43 (HS43) and hydroponic traits. (DOCX 12 kb)

Additional file 10: Table S5. Mean rhizotron data for 12 accessions. (XLSX 14.4 kb)

Additional file 11: Table S7. Correlations between herbicide scores day 43 (HS43) and rhizotron traits. (DOCX 12 kb)
