# Supplementary figures and images for: Genetic and root phenotype diversity in Sri Lankan rice landraces may be related to drought resistance
Source: Rice (N Y). 2016 May 17;9:24. doi: 10.1186/s12284-016-0092-7 (PMC5396129; doi:10.1186/s12284-016-0092-7)

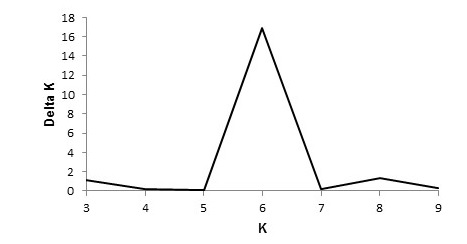

Supplement: Supplementary file 1 — Optimum K value of 6 revealed in the K vs delta K plot from STRUCTURE. (DOCX 24 kb) [file 12284_2016_92_MOESM1_ESM.docx]

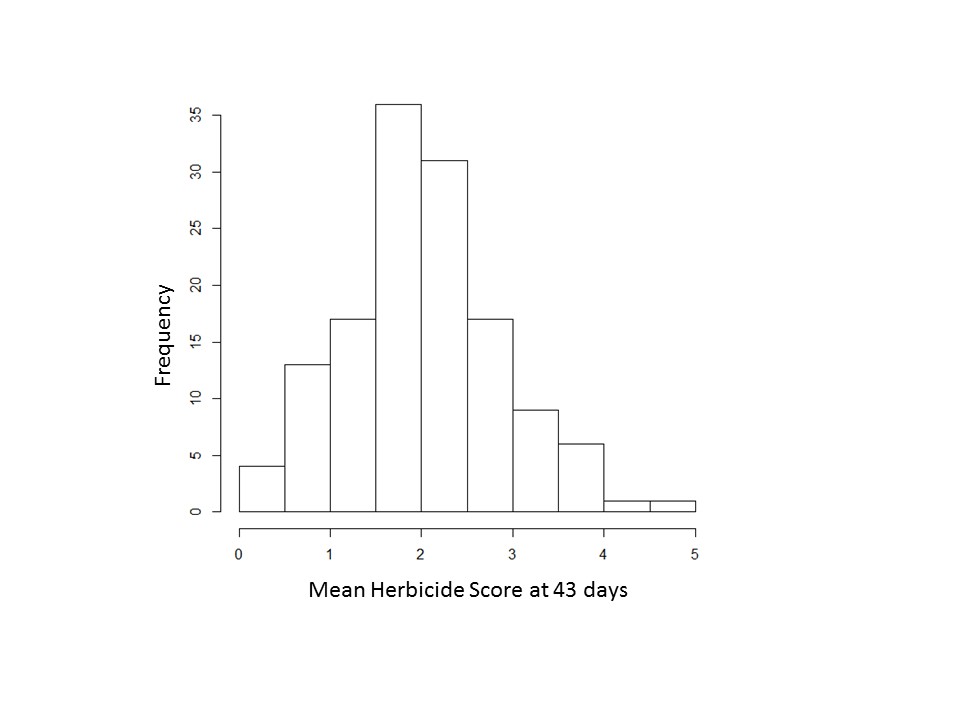

Supplement: Supplementary file 7 — Frequency distribution of mean herbicide score at 43 days for 135 Sri Lankan landraces. (DOCX 43 kb) [file 12284_2016_92_MOESM7_ESM.docx]
