# Supplementary material for: Genetic and root phenotype diversity in Sri Lankan rice landraces may be related to drought resistance
Source: Rice (N Y). 2016 May 17;9:24. doi: 10.1186/s12284-016-0092-7 (PMC5396129; doi:10.1186/s12284-016-0092-7)
Supplement: Supplementary file 6 — Correlations between herbicide scores (HS) at different days. (DOCX 12 kb) [file 12284_2016_92_MOESM6_ESM.docx]

**Supplementary Table 3**- Correlations between herbicide scores (HS) at different days

|  | HS21 | HS26 | HS31 | HS37 |
| --- | --- | --- | --- | --- |
| HS26 | 0.479 |  |  |  |
| HS31 | 0.370 | 0.863 |  |  |
| HS37 | 0.390 | 0.804 | 0.935 |  |
| HS43 | 0.375 | 0.788 | 0.872 | 0.920 |

All correlations significant at P < 0.001.
