# Supplementary material for: Genetic and root phenotype diversity in Sri Lankan rice landraces may be related to drought resistance
Source: Rice (N Y). 2016 May 17;9:24. doi: 10.1186/s12284-016-0092-7 (PMC5396129; doi:10.1186/s12284-016-0092-7)
Supplement: Supplementary file 9 — Correlations between herbicide score day 43 (HS43) and hydroponic traits. (DOCX 12 kb) [file 12284_2016_92_MOESM9_ESM.docx]

**Supplementary Table 6-** Correlations between herbicide score day 43 (HS43) and hydroponic traits

| Trait | HS43 |
| --- | --- |
| Max. root length day 7 | 0.520*** |
| Max. root length 14 | 0.678*** |
| Max. root length 21 | 0.732*** |
| Max. root length 28 | 0.744*** |
| Max. root length 35 | 0.706*** |
| Root dry weight | 0.639** |
| Root thickness | 0.323 |
| % Root mass | -0.163 |
| Shoot length day 7 | 0.283 |
| Shoot length 14 | 0.681*** |
| Shoot length 21 | 0.331* |
| Shoot length 28 | 0.380* |
| Shoot length 35 | 0.587*** |
| Shoot dry weight | 0.584*** |
| Plant dry weight | 0.595*** |

* P < 0.05; ** P < 0.01; *** P < 0.001
