# Supplementary material for: Genetic and root phenotype diversity in Sri Lankan rice landraces may be related to drought resistance
Source: Rice (N Y). 2016 May 17;9:24. doi: 10.1186/s12284-016-0092-7 (PMC5396129; doi:10.1186/s12284-016-0092-7)
Supplement: Supplementary file 11 — Correlations between herbicide scores day 43 (HS43) and rhizotron traits. (DOCX 12 kb) [file 12284_2016_92_MOESM11_ESM.docx]

**Supplementary Table 7-** Correlations between herbicide scores day 43 (HS43) and rhizotron traits

| Trait | HS43 |
| --- | --- |
| Max. root length day 7 | 0.675* |
| Max. root length 14 | 0.842*** |
| Max. root length 21 | 0.883*** |
| Max. root length 28 | 0.857*** |
| Max. root length 35 | 0.779** |
| Max. root length 42 | 0.733** |
| Root dry weight | 0.831*** |
| Root thickness | 0.784** |
| % Root mass | 0.629* |
| Root angle day 21 | -0.719** |
| Root angle day 42 | -0.901*** |
| Average main axis angle | 0.623* |
| Shoot length day 7 | 0.524 |
| Shoot length 14 | 0.603* |
| Shoot length 21 | 0.740** |
| Shoot length 28 | 0.678* |
| Shoot length 35 | 0.748*** |
| Shoot length 42 | 0.718** |
| Shoot dry weight | 0.752** |
| Plant dry weight | 0.783** |

* P < 0.05; ** P < 0.01; *** P < 0.001
